# Dr. William Harris

**Published:** 1861-05

**Authors:** 


DR. WILLIAM HARRIS, a brother of Dr. Thomas Harris, and long known in this city as an excellent practitioner, died at his residence on Spruce Street, on the 3d of March, also of apoplexy, in the sixty-eighth year of his age. He had been out visiting a patient two days before, when he was suddenly seized with paralysis and unconsciousness, which continued up to the moment of his dissolution. Dr. Harris was for a time a private teacher of obstetrics in this city. He contributed several valuable papers to the periodical press, and was the author of a translation of Aran's work on the Diseases of the Heart.

